# Towards people-centred health services delivery: a Framework for Action for the World Health Organisation (WHO) European Region

**DOI:** 10.5334/ijic.1514

**Published:** 2013-12-23

**Authors:** K. Viktoria Stein, Erica Stukator Barbazza, Juan Tello, Hans Kluge

**Affiliations:** Division of Health Systems and Public Health, WHO Regional Office for Europe, Denmark

Integrated care has moved from the small niche it traditionally occupied in academia, accessible only to experts in the field and applied merely on a project specific or pilot effort basis, now to the radar of politicians and health system planners the world over.

More than a buzzword for the 21st century, coordinated/integrated health services delivery is a necessity. From changing demographics and increasing chronicity to the persisting threat of communicable diseases, coupled with modern technologies, rising patient expectations and a perpetual context of fiscal constraints, new and innovative approaches to the delivery of health care, that ensure high-quality services which are efficient in their provision and delivered according to an individual's needs, must be given the utmost priority.

Strengthening the coordination/integration of care is ultimately best viewed as a means, rather than an end in itself, for improved health outcomes. The activity in this area can be credited to this presumed causal relation. The vast number of initiatives to design and implement integrated care programmes across countries in the WHO European Region is astounding. These examples extend from local initiatives like integrated care pilots in North West London in the United Kingdom [1] to regional efforts seen in Lithuania and Ukraine to strengthen disease management, and on the largest scale, national efforts like that of the Basque Strategy to tackle chronicity in Spain [2], or the Croatian eHealth strategy paving the way for coordinated communication flows [3].

Still, there remains a long way ahead to transform services to people-centred care: lack of clearly defined and measurable aims, consistent communication strategies or participatory approaches in developing and implementing integrated care are only a few of the shortcomings that may lead to sub-optimal outcomes or present challenges in order to implement efforts sustainably and at scale. Ensuring system-wide implementation is often further hampered by ambiguous incentive structures, a lack of adequately trained professionals and/or outdated legal frameworks.

The variability in approaches is in itself a testament to the diversity and flexibility of integrated care – a characteristic that is also its strong point, as integrated care has never been intended as a mere theoretical concept, but was rather developed *from* practice and *for* practice. Reforming a health system to meet the challenges of the 21st century is no small feat and it is only with the many different attempts to integrating care that we have come to understand the complexities of health system. As Goodwin recently acknowledged [4], there is a growing interest in ‘what works, how, where and why’, and there are already some very impressive examples of having gotten integrated care ‘right’ [1,5].

As the interest for integrated care has grown, so has the attention of governments, the World Health Organization, the European Union and other international institutions in this topic. At the WHO Regional Office for Europe, attention is drawn to the vision of Health 2020 [6] for improving health system performance through innovative approaches to modernise and transform the delivery of health services towards more people-centredness. The newly founded WHO Centre for Primary Health Care in Kazakhstan will promote the idea of people-centred care, advocating a proactive primary health care setting and will create a platform for capacity-building and knowledge exchange in the region.

Just this past October, the WHO Regional Office for Europe officially launched the roadmap to develop a Framework for Action towards coordinated/integrated health services delivery at the 5th Tallinn Charter Anniversary WHO High-Level Meeting in Tallinn, Estonia. This framework follows the call from Member States for contextualised, evidence-based policy options to enable system-wide changes and the need for tools to implement these changes. The framework is aligned with other WHO global initiatives in this area and involves a wide range of stakeholders including national representatives from countries across Member States and international experts, among others, to solicit their input through various consultations and feedback loops. The way forward was defined in the aforementioned roadmap document, which outlines three pillars for action. These pillars correspond to three topical questions:

1. **What is *coordinated/integrated health services delivery* and how can it be operationalised to lead to health system strengthening?**
  This will be responded to within a concept note, synthesising the existing literature and evidence, and outlining WHO Europe's definition of coordinated/integrated health services delivery as the design, management and delivery of health services such that people receive and perceive a continuum of health promotion, health protection and disease prevention services, as well as diagnosis, treatment, long-term care, rehabilitation and palliative care services through the different levels and sites of care within the health system and according to their needs. The uniqueness of WHO's mandate permeates this definition, which includes public health as a vital part of the health system, and puts people, not necessarily patients, into the centre of attention. In operationalising this definition, intersectoral actions towards including social services, educational sector and legal frameworks are needed throughout the integration process.
2. **What are the experiences of Member States with implementing more integrated care and how do the lessons from these implementation efforts apply to other contexts?**
  A second pillar collects experiences from across the WHO European Region, with its 53 member states, by means of an open call for initiatives and in-depth country case studies. While the former will draw a picture of what is already happening and where, the latter will identify the arenas for action necessary to create sustainable and equitable systems change towards coordinated/integrated health services delivery. As outlined above, successful examples like the Eastern Lithuanian Cardiology Programme [7], the ‘Healthcare Quality Strategy for National Health Services Scotland’ [8] or the Ukrainian integrated care programme for HIV/TB [9] already show us the way forward, and it is the aim of this Framework for Action to highlight these stories, as well as lessons learned.
3. **How can policy-makers, providers, patients and communities lead and manage transformations towards more** coordinated/integrated health services delivery?
  Finally, the case studies and country experiences, as well as the existing knowledge captured in the concept note, will inform recommendations and policy options to manage and implement sustainable change. By creating an environment conducive to innovative approaches, it is anticipated that a change in values and cultures will also take place, from the primary care setting across the boundaries to hospitals and long-term care facilities, in order to overcome barriers and enable a truly holistic approach towards people-centred services.

Challenging the status quo is never easy. But as our Member States have shown, changing the delivery of care to provide more coordinated/integrated health services is a needed effort towards high-quality, sustainable, people-centred health systems. And it can be done. The momentum this agenda has garnered is merited to its applicability given the current context of health and society. Nevertheless, much work remains. We must continue to expand our thinking and experiences in this field, overcoming limitations of small-scale efforts, responding to concerns of sustainability, and ultimately, reaching a level of sophistication that enables the coordination/integration of services to be truly tailored to the individual and not restricted, for example, by geography, disease types or privileged groups of society. That is, in continuing to transform our health services towards more coordinated/integrated care, ultimately, each nation, region, community, family and individual may realise their full health potential.

The WHO Regional Office for Europe new health policy, Health 2020 [6], calls for a whole-system and whole-society-approach to meet the challenges of the 21st century and create more people-centred health systems. These transformations call for a paradigm shift in thinking health, actively involving people to participate in the organisation of health systems, and strengthening communities to create healthy environs. In order to support these efforts in the Member States, the Health Services Delivery Programme (HSD) in the Division of Health Systems and Public Health (DSP) of the WHO Regional Office for Europe initiated the development of a Framework for Action towards CIHSD. From 2013 until 2016, this participatory process will synthesise the evidence on integrated care delivery and change management, gather field evidence across the WHO European Region and design a toolkit for sustainable system transformation. Along this way, active participation of the Member States, experts, patients and provider organisations is solicited to meet the principles set forth in Health 2020 [6].

*Note*: As a first effort to consolidate experiences, we invite the exchange of practices to create more coordinated/integrated services from across the WHO European Region through an open call for initiatives. Whether a local effort to minimise fragmentation of care in a given centre, or a regionally or nationally planned initiative linking providers across sites and levels of care – whichever the scale, shape and form – your support in capturing these efforts is needed. For more information on this open call for practices and to fill in the simple electronic questionnaire to do so – as a manager, policy-maker, provider or participant in an initiative – we invite you to visit our website at: www.euro.who.int/en/what-we-do/health-topics/Health-systems/health-service-delivery

English:

http://www.euro.who.int/en/health-topics/Health-systems/health-service-delivery/news/news/2013/11/call-for-submission-of-initiatives-towards-the-coordinationintegration-of-health-services-delivery-cihsd-in-the-who-european-region/_recache

Russian:

http://www.euro.who.int/ru/health-topics/Health-systems/health-service-delivery/news/news/2013/11/call-for-submission-of-initiatives-towards-the-coordinationintegration-of-health-services-delivery-cihsd-in-the-who-european-region/_recache

To send questions, feedback or input, please contact cihsd@euro.who.int
